# The epidemiological status of urogenital schistosomiasis among reproductive aged individuals in the Tiko Health Area- a semi-urban setting in the Mount Cameroon area

**DOI:** 10.1371/journal.pntd.0008978

**Published:** 2021-01-11

**Authors:** Vicky Daonyle Ndassi, Judith Kuoh Anchang-Kimbi, Irene Ule Ngole Sumbele, Lennin Azaofah Ngufor, Kouemou Nadege, Helen Kuokuo Kimbi

**Affiliations:** 1 Department of Zoology and Animal Physiology, Faculty of Science, University of Buea, Buea, Cameroon; 2 Department of Medical Laboratory Science, Faculty of Health Sciences, University of Bamenda, Bamenda, Cameroon; PUCRS, BRAZIL

## Abstract

**Background:**

Urogenital schistosomiasis (UGS) caused by *S*. *haematobium* has enormous reproductive health consequences including infertility. Reproductive aged individuals are a neglected group and not included in control programs in Cameroon. This study investigated the prevalence and severity of *S*. *haematobium* infection in the context of gender and socio-economic structures that shape behaviour among reproductive aged individuals living in Tiko, a semi-urban setting, Cameroon.

**Methodology/Principal findings:**

A cross-sectional study was carried out in the Tiko Health District (THD) between May to September 2019. Consenting individuals were enrolled using a convenient sampling technique and administered a semi-structured questionnaire to document data on socio-demographic and stream contact behaviour. A urine sample was collected and screened for the presence of *S*. *haematobium* ova using reagent strips, filtration and microscopy. The overall prevalence of *S*. *haematobium* infection was 22.8% (95% CL: 19.27–26.73) with geometric mean egg load of 18.74 (range: 1–1600) per 10ml of urine. Younger age group (15 – 20years) (OR: 5.13; 95% CL: 1.35–19.42), male (OR: 2.60 3.07; 95% CL: 1.54–4.40) and awareness of UGS (OR: 1.73; 95% CL: 1.02–2.95) were associated with higher odds of exposure to infection. Significantly higher intensity of infection was seen in males, singles and in the age group 15–30 years. It is worth noting that males carried out more activities which entailed longer duration in streams.

**Conclusion/Significance:**

The prevalence obtained shows that Tiko is a moderate-risk area for UGS with underlying morbidity-inducing infection intensity. The severity of the infection is more in males. Awareness of the disease is not enough to protect these communities from infection, but provision of public infrastructures and health education will limit contact with infested water and thus curtail the infection. There is an urgent need to involve all age groups in control programs.

## Author summary

*S*. *haematobium* deposit its eggs in the urogenital organs which may cause genital ulcers and other lesions. When untreated the infection causes infertility, miscarriage, ectopic pregnancies, spontaneous abortions, prematurity, vulva nodules, genital and cervical lesions, increased risk of HIV and Human Papilloma Virus in women. These may lead to social consequences such as low self-esteem, depression and stigma. The disease burden has been neglected in males, with more emphasis on female genital schistosomiasis. Control programs have focused on school age children, often overlooking reproductive aged individuals who are potential reservoirs. A total of 509 reproductive aged individuals living in Tiko, a semi urban setting in the mount Cameroon area were screened. The overall prevalence of *S*. *haematobium* infection was 22.8%, with significantly higher rates in males (35.2%) than females (16.2%). Similarly, intensity of infection was significantly higher in males (p = 0.009), singles (p = 0.004) and younger age individuals (0.012). Younger age, gender type (male) and awareness of UGS were identified as key factors that increase the odd of infection. In this setting, younger adults and males represent important public health risk groups and potential reservoir sources of disease transmission in the area.

## Introduction

Schistosomiasis is an acute and chronic disease caused by dioecious blood flukes of the genus *Schistosoma*. It remains a major neglected tropical disease (NTD) and a significant public health challenge in low and middle-income countries [1]. It is estimated that at least 90% of those requiring treatment for schistosomiasis live in Africa where two forms of schistosomiasis (intestinal and urogenital) exist [2]. Schistosomiasis mostly affects poor and rural communities; however, migration to urban areas and population movements are introducing the disease to new areas [2]. The transmission of schistosomiasis is governed by social-ecological systems such as conditions of poverty and living near open freshwater bodies [3]. In endemic areas, where there is lack of adequate water supply, poverty, ignorance, and poor hygienic practices, any demographical groups, irrespective of age or gender with unsafe water contact is at risk of infection [4–8]. Nonetheless, key gendered roles and customs place men, women, girls and boys at differential risk. The roles of women and girls within the household including chores such as washing of clothes and dishes, collecting water for household consumption exposes them to daily risk of infection [9, 10]. Livelihood activities such as fishing, agriculture which often require contact with infested waters have been linked to the male gender [10].

Urogenital schistosomiasis (UGS) is caused by infection with *S*. *haematobium*. People become infected when the larval form, the cercaria, released by freshwater snails of the genus *Bulinus* penetrate the skin during contact with infested water. In the body, the adult female fluke lay terminal-spined eggs, often in copious amounts each day. Some of the eggs are passed out of the body in urine to continue the parasite’s lifecycle while others become partially lodged or later trapped within all organs of the urogenital tract causing immune reactions and progressive damage to organs [2]. In the bladder, the eggs perforate and cross the bladder wall with accompanied leakage of venous blood. This leads to the classical sign of UGS known as haematuria. Fibrosis of the bladder and ureter, kidney damage and possibly bladder cancer are sometimes complications in advanced cases [11, 12]. In adults, the infection can cause genital ulcers and other lesions [13] resulting in long-term poor reproductive health, with sexual dysfunction and irreversible consequences including infertility [14]. Urogenital schistosomiasis is also considered to be a risk factor for HIV infection [15, 16] and Human Papilloma Virus in adults [1]. The consequences and disability caused by gender specific manifestations of UGS often go unrecognized at national and local levels. Also, unlike female genital schistosomiasis (FGS), male genital schistosomiasis (MGS), as evidenced by schistosome eggs in male genital organs remains underreported and often misperceived [16].

Typically, UGS is endemic in rural areas of the Bafia Health Area [17–19] found in the Mount Cameroon area. In 2018, an unmapped UGS transmission focus was reported in Tiko, a semi urban town in the THD, Mount Cameroon Area, probably due to human migration and interurban trading which occurs between rural and peri- urban settings in the area [20]. Moreover, the equatorial climate contributes to establishment of the infection because it provides conditions suitable for the presence of the molluscs that encourage the transmission of this disease in this endemic area [21]. While the WHO strategy recommends reaching both school-based and community-based programs, the national control programmes in Cameroon prioritize mass drug administration of praziquantel to school-age children [22]. Adults excluded from preventive chemotherapy campaigns continue to potentiate transmission in endemic communities. Praziquantel based control programs have only a temporary effect on transmission and are limited in their potential to interrupt disease transmission in the long-term [23]. More so, untreated infected individuals may suffer from profound adverse urothelial and reproductive health consequences. A global analysis by Kayuni *et al*. [16] revealed the existence of a gap in epidemiological data on the MGS and FGS in Cameroon. This suggests that the burden of disease in reproductive aged individuals is not fully recognized probably due to lack of disease monitoring by health systems in endemic communities. This study aims to provide an update on epidemiological findings on UGS among reproductive aged individuals living in the Tiko Health District, Cameroon. The prevalence and severity of *S*. *haematobium* infection was evaluated in the context of gender and socio-economic structures that shape behaviour. Identifying local risk factors is essential for expediting disease control by targeting high-risk groups or by informing possible intervention strategies to stakeholders involved in the control of schistosomiasis.

## Materials and methods

### Ethics statement

The study received institutional approval (2017/645-08/UB/SG/IRB/FHS) from the Ethics Review Board hosted by the Faculty of Health Sciences and administrative authorisation from the South West Regional Delegation of Public Health, Buea. All participants were invited to sign the free and informed consent form. In this document, participants must have the freedom to leave the study at any time. For participants less than 18 years old, consent was gotten from the parents or guardian and the child meanwhile children 18 years old and older individuals gave their consent. Discussions were made in the local language (pidgin) when necessary and simplified to the best understanding of the participants. By signing the form, the participants agreed to be administered a questionnaire and to provide urine sample for the parasitological analysis.

The results were communicated to the participants, and adults positive for *S*. *haematobium* infection were treated for free with praziquantel tablets (40 mg/kg of body weight) (Cesol 600mg manufacture by Merck, Mexique). The treatment was in accordance with Cameroonian guidelines of the Control Program of Schistosomiasis (PNLSHI).

### Study area

**Fig 1:**
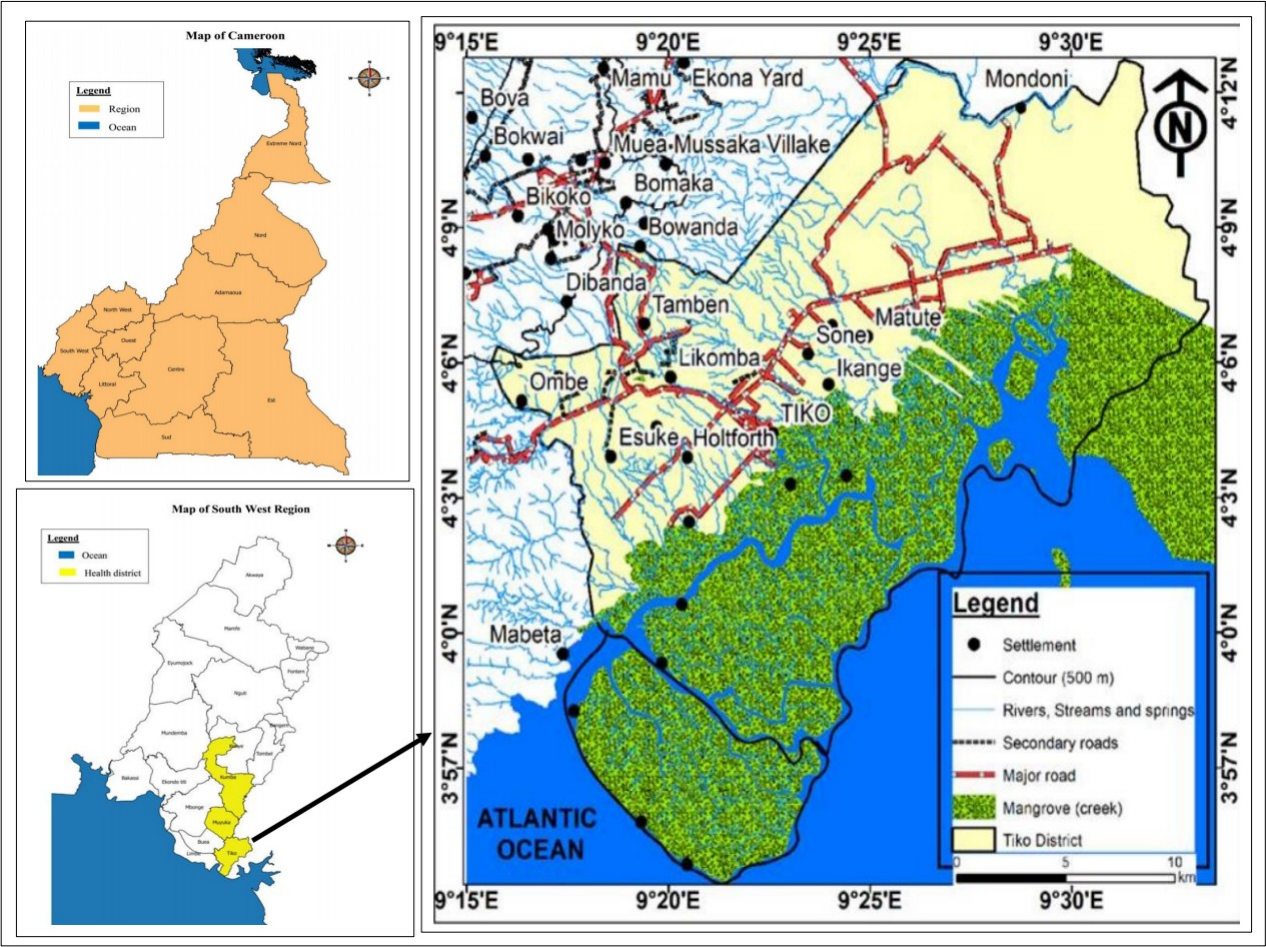
Map showing Tiko Health District in the Mount Cameroon area.

This study was carried out in the Tiko Health Area, an unmapped endemic foci [20], which is geographically close to Munyenge, a rural endemic foci [17, 19], located in Muyuka health district, Mount Cameroon area, South West Region, Cameroon Fig 1. Tiko town is a semi-urban settlement with a surface area of 4840 Km^2^ and a population size of 134,649. The population density of Tiko is 241 inhabitants/Km^2^, with a population growth of 2.9% [20]. Tiko is located 10 meters above sea level between longitudes 9°15′E and 9°30′E, latitudes 3°57′N and 4°12′N with a relative humility of 83.1% and average rain fall of 4,524 mm. This area has a coastal equatorial climate with daily temperatures ranging from 28°C—33°C. Soil types include the sandy alluvial and volcanic with high agricultural potentials. The Tiko municipality is interspersed with water courses including rivers Mungo and Ombe, and Ndongo and Benyo streams which empty into the Atlantic Ocean [24] (Fig 1). The predominant livelihoods of people living in the communities are trading, farming and fishing [24]. For some communities with deficiency in water supply, accessing and using stream water is a vital part of both household survival and many livelihood activities such as farming (washing pumpkin seeds). The lack of bridges in certain parts of the town, compels the population crossing these streams to their farms and other areas within the municipality exposing them to cercariae infested streams. The study was carried out in four communities (Likomba, Holtfort, Tiko, Ikange) in the Tiko Health Area which are in proximity to the streams.

### Study participants and sample size determination

The study population included reproductive aged individuals (females: 15–49 years, Male: 15–71 years, as specified by Yirenya-Tawiah *et al*. [25] and Marlowe *et al*. [26]) living in the Tiko Health District. The sample size of the study population was calculated considering *S*. *haematobium* infection prevalence of 38% from a recent cross sectional survey carried out in the Tiko Health District by Anguh *et al*. [20]. The sample size was determined using the Lorenz formula [27]. The total number of samples N is given by: N = Z^2^ P (1-P)/d^2^ where Z is the standard normal deviation, Z = 1.96 for the confidence level of 95%, P = 38%: proportion of UGS prevalence, d is the total width of the confidence interval (e.g., 0.05 = ±5). The minimum estimated sample size calculated was 362. A total of 514 participants was enrolled into this study to anticipated factors like effect size, loss of data, voluntary withdrawal and for greater precision.

### Study design

To evaluate the prevalence and severity of *S*. *haematobium* infection among individuals of reproductive age in the study area, a cross-sectional study was conducted between May and September 2019. After obtaining administrative and ethical clearances, acquaintance visits were made to community and church leaders of the communities concerned, to inform them about the study procedures. Questionnaire was pre-tested by research assistants, challenges were identified and the changes effected. A convenience sampling technique was used to recruit participants into the study. Individuals were invited by local coordinators and community leaders to rally at the different church premises and focal points in the four selected communities on the day programmed for data and sample collection. Health talks were given in layman’s terms, to explain the purpose, risk and benefits of the study. A semi-structured questionnaire was administered to volunteer participants by trained research assistants (including the first and fourth authors) to obtain data on sociodemographic and economic factors as well as stream contact behaviour. Discussions were made in English and the local language (pidgin) where necessary and simplified to the best understanding of the participants. Urine samples were collected and processed for the detection of haematuria and *S*. *haematobium* ova.

### Inclusion, exclusion and withdrawal criteria

This study was designed to target individuals of reproductive age (females: 15–49 years, Male: 15–71 years). Only respondents who have lived for at least two months in the study area and volunteered to participate were enrolled in the study. Those who declined to participate in the study or failed to submit a urine sample after the interview were excluded from the survey.

### Data collection

#### Administration of questionnaire

A semi-structured questionnaire (S1 Checklist Questionnaire) was used to interview the participants face to face to obtain information about individuals based on the following indicators: socio- demographic/economic (resident address, distance from stream, age, gender, educational level, marital status, occupation) and water contact behaviour (source of water supply, stream usage, frequency of contact with open water source, and activities carried out in the stream among other information). The degree of water contact was calculated using the formula: Σ(R x F) as described by Lima *et al*. [28], R is the score for the reason for the contact and F the score for the frequency of contact. The reasons for water contact are given the following scores: 5 (bathing), 4 (laundry, washing of motorbikes), 3 (collecting water for the household washing, washing pumpkin seeds in the stream), and 2 (crossing the streams). The frequency of contacts were scored according to Lima *et al*. [28] with some modifications: 28 (more than thrice a week or daily), 12 (three contact a week), 8 (two contact a week), and 4 (one contact a week). Totals of ≥100 were considered as high degree and 2–99 as low degree [28].

#### Sample collection and laboratory analysis

On the day of enrolment, all individuals who participated in the interviews received a sterile, wide mouthed, screw capped plastic bottle carrying their identification information. Due to the circadian pattern of schistosome egg excretion, participants were requested to collect a urine sample between 10 am– 2 pm [29]. All samples were stored in cooling boxes containing air cooler ice packs at temperature of about 3.7°C to prevent the eggs of *S*. *haematobium* from hatching during transportation to the THD laboratory for processing within 24 hours of collection. In the laboratory, haematuria was immediately determined by visual observation and urine reagent strips (Mission* Expert USA). The urine samples were processed using the membrane filtration technique and examined microscopically for the presence of *S*. *haematobium* infection based on morphology of the ova [30]. In brief, 10 ml of urine was pass through membrane filter (Sterlitech Polycarbonate (PCTE) membrane filters, USA), the filter was removed, place on a glass slide and stained with 1% Lugol iodine solution. The slide was then examined using the Binocular Compound light microscope. Terminal-spined eggs, characteristics of *S*. *haematobium* were identified and counted manually [30]. The egg load was defined by the number of eggs per 10ml of urine, categorized as light (<50 eggs/10 ml of urine) or heavy (≥50 eggs/10ml of urine) infection as defined by the WHO [31]. Microhaematuria was considered as proxy-diagnosis of UGS, an accepted marker in the rapid diagnosis of *S*. *haematobium* infection in urine [32]. An individual was considered positive for UGS, when he or she had *S*. *haematobium* eggs and/or positive for microhaematuria.

### Statistical analysis

The data were analysed using SPSS version 21.0 (SPSS, Inc., Chicago, IL, USA). Proportions of *S*. *haematobium* infection were compared between different groups (age groups, sex, educational level, occupational status, distance of house from stream, stream usage, stream contact activities, and frequency to stream) using Pearson Chi-square test (χ^2^). Worm egg output was normalized by log_10_ transformation and assessed in relation to age using Pearson’s correction coefficient (r). Kruskal Wallis and Mann-Whitney tests were used to compare mean differences in the intensity of egg excretion. Odd ratios (OR) and confidence intervals (CIs) were calculated using a Microsoft Excel confidence interval calculator as described by Armitage & Berry [33] and Newcombe [34]. Variables that had a p-value <0.20 in bivariate analysis or explanatory plausibility were included in the logistic regression model for analysis of factors associated with *S*. *haematobium* infection. A p—value of < 0.05 was considered statistically significant.

## Results

### Characteristics of the study population

In this study, a total number of 509 individuals of reproductive age (range: 15–71 years) were enrolled, completed questionnaires, and submitted urine samples. The mean age was 32.3 ± 11.3 years and 65.4% were females. Of noteworthy, 83% have lived in the study area for more than 4 years and only 31.8% of the participants have heard of urogenital schistosomiasis, 64.5% live within 100m away from infested streams among whom almost two-third rely on stream water (66.4%, n = 338). The principal stream activities reported are bathing, laundry, collection of water for domestic activities, farming and washing of motorbikes. Overall, 33.6% reported use of tap water only and were mostly those who live more than 100m from infested water sources. All the participants have basic level of education, 58.2% of them having obtained some form of secondary education. The characteristics of the study population are shown in Table 1.

**Table 1:** Characteristics of the study participants.

Variable	Characteristics	Number examined (N)	Percentage (%)
Gender	Female	333	65.4
	Male	176	34.6
Age Group (Years)	15–20	109	21.4
	21–30	114	22.4
	31–40	160	31.4
	41–71	126	24.8
Marital status	Single	230	45.2
	Married	279	54.8
Educational level	At least Primary	213	41.8
	At least Secondary	296	58.2
Occupation	Student	117	23.0
	Farmer	119	23.4
	Business	133	26.1
	Salary earner	140	27.5
Distance to stream (metres)	≤ 100	329	64.6
	> 100	180	35.4
Awareness	Aware	162	31.8
	Not aware	347	68.2
Source of water	Stream only	64	12.6
	Stream and piped	274	53.8
	Piped only	171	33.6
Stream usage	Yes	338	66.4
	No	171	33.6
Stream Activity	Bathing	166	29.0
	Laundry	189	33.0
	Fetch water	138	24.0
Frequency to stream per week	≥ Thrice	199	58.9
	< Thrice	139	41.1

### The prevalence and intensity of *S*. *haematobium* infection and associated determinant factors

**Fig 2:**
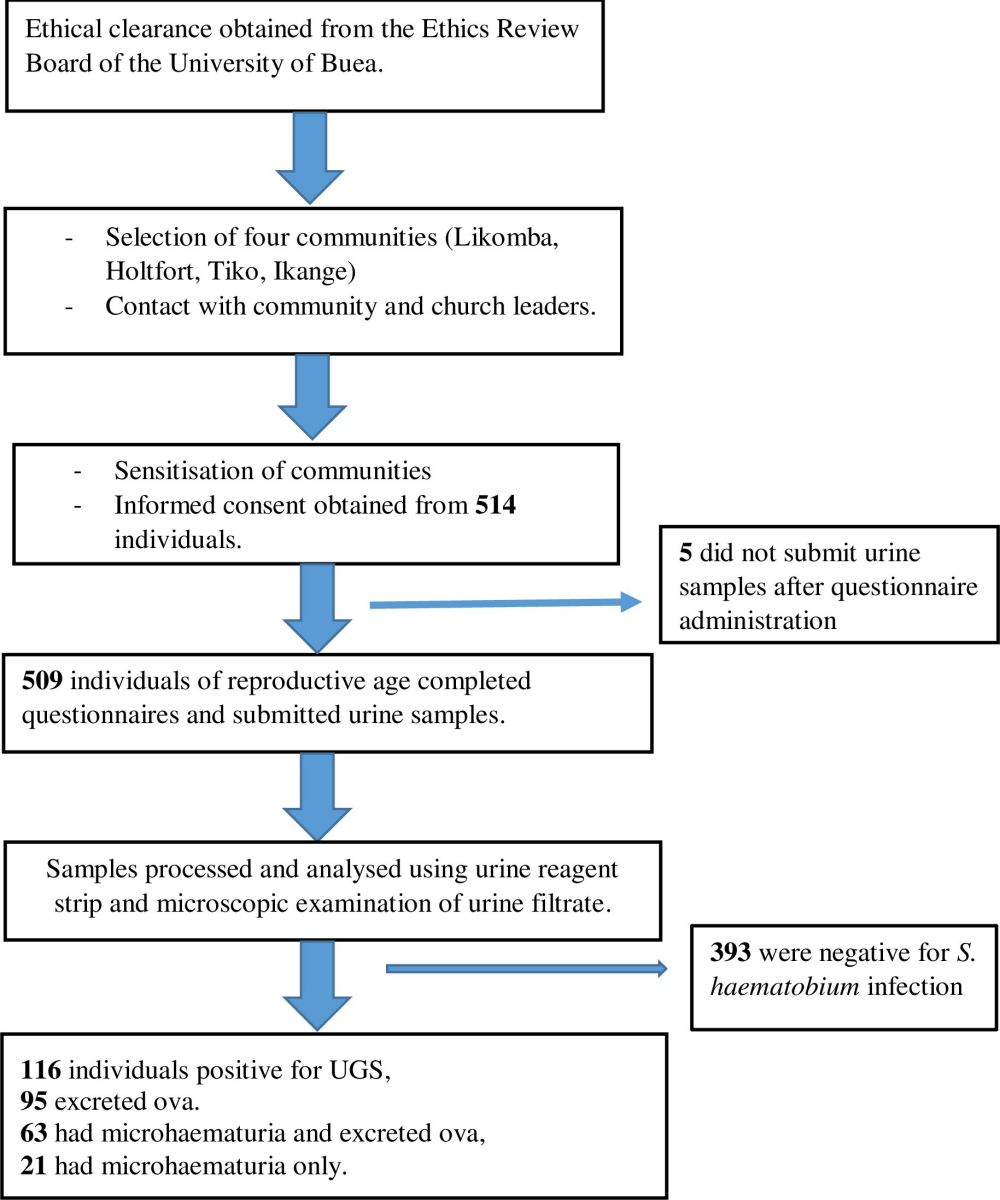
Flow chart of participation and prevalence of urogenital schistosomiasis.

In this study, 116 (22.8%; 95% CI: 19.27–26.73) individuals were positive for UGS. Egg excretion was recorded for 95 (18.7%) among whom 28 (29.5%) had heavy (≥50 eggs/10ml of urine) infection while 67 (70.5%) had light (<50 eggs/10ml of urine) infection. The geometric mean load was 18.74 (range: 1–1600) eggs per 10ml of urine. The prevalence of microhaematuria was 12.4% (n = 63), of which 4.1% (21) were positive for microhaematuria only Fig 2. Using microscopic urine examination as gold standard, the specificity and sensitivity of microhaematuria in the diagnosis of *S*. *haematobium* infection were 95.0% (95% CI: 92.4–96.7) and 44.2% (95% CI: 34.6–54.2), respectively.

The results displayed in Table 2 shows that age (p <0.001), gender (p <0.001), marital status (p <0.001), secondary level of education (p = 0.017), the type of occupation (p <0.001), distance from house to stream (p = 0.004), source of water (p <0.001) and activity at stream were the determinant factors of urogenital schistosomiasis in the study area. Accordingly, higher values of prevalence were obtained among; the younger age group (15–20years) (45.9%), males (35.2%), individuals who had attained at least secondary level of education (26.7%), singles (31.7%) and students (41.9%). In addition, a higher prevalence was recorded among participants who reported using the stream as the only source of water (42.2%), washing of motorbike in the stream (75%) and those who live within 100m away from infested streams (27.7%).

**Table 2:** Univariable analysis of *S*. *haematobium* infection according to sociodemographic and behavioural factors.

Variable	Category	*S*. *haematobium* Positive % (n)	χ2; p value
Gender	Male	35.2 (62)	23.650; < 0.001
	Female	16.2 (54)	
Age group(years)	15–20	45.9 (50)	42.509; < 0.001
	21–30	17.5 (20)	
	31–40	17.5 (28)	
	41–71	14.3 (18)	
Educational level	At least secondary	26.7 (79)	6.112; 0.013
	At least primary	17.4 (37)	
Marital status	Single	31.7 (73)	19.099; <0.001
	Married	15.4 (43)	
Occupation	Student	41.9 (49)	35.768; <0.001
	Farming	21.8 (26)	
	Business	18.8 (25)	
	Salary earner	11.4 (16)	
Awareness	Aware	27.8 (45)	3.360; 0.067
	Not aware	20.5 (71)	
Distance from stream (metres)	≤ 100	27.7 (91)	12.539; < 0.001
	˃ 100	13.9 (25)	
Source of water	Stream only	42.2 (27)	49.931; < 0.001
	Stream and piped	29.2 (80)	
	Piped only	5.3 (9)	
Stream activity	Washing motorbike	75.0 (6)	23.846; < 0.001
	Bathing	34.9 (58)	
	Laundry	34.6 (65)	
	Fetch water	33.3 (46)	
	Farming	25.0 (18)	
Frequency to stream per week	≥ Thrice	33.2 (66)	0.509; 0.475
	< Thrice	29.5 (41)	

χ^2^ = Pearson chi-square test.

In Table 3, the binary logistic model presents determinant factors associated with the risk of *S*. *haematobium* infection. The most important factors associated with infection in the study area were age and gender. Age groups 15–20 years and 31–40 years were found to be 5 times (95% CI: 1.35–19.42) and 2 times (95% CI: 1.08–5.18) more likely to be infected compared with others in their respective categories. In similarity to the odd of infection, male respondents were observed to be about 3 times (95%CI: 1.54–4.40) more at risk of being infected with the cercariae of *S*. *haematobium* when compared with their female counterparts Table 3. It is puzzling that individuals who were aware of urogenital schistosomiasis were almost two times (95%CI: 1.02–2.95) more likely to be infected with *S*. *haematobium* when compared with those who had not heard of the disease. Of noteworthy, out of the 162 respondents aware of UGS, 116 (71.6%) live within 100m away from infested open water sources compared with those who live at the distance of more than 100m (28.4%; n = 46). The difference was statistically significant (χ^2^ = 5.05; p = 0.025).

**Table 3:** Risk factors associated with urogenital schistosomiasis among reproductive aged individuals in Tiko.

Variable	Category	*S*. *haematobium* Positive % (n)	Unadjusted OR (95% CI)	^#^Adjusted OR (95% CI)	P- value
Gender	Male	35.2 (62)	2.81 (1.84–4.30)	2.60 (1.54–4.40)	< 0.001
	Female	16.2 (54)	1.00	1.00	
Age group(years)	15–20	45.9 (50)	5.08 (2.72–9.50)	5.13 (1.35–19.42)	0.016
	21–30	17.5 (20)	1.28 (0.64–2.56)	1.50 (0.58–3.86)	0.403
	31–40	17.5 (28)	1.27 (0.67–2.42)	2.37 (1.08–5.18)	0.031
	41–71	14.3 (18)	1.00	1.00	
Educational level	At least secondary	26.7 (79)	1.73 (1.12–2.68)	1.18 (0.62–2.25)	0.616
	At least primary	17.4 (37)	1.00	1.00	
Marital status	Single	31.7 (73)	2.55 (1.66–3.91)	0.77 (0.37–1.60)	0.482
	Married	15.4 (43)	1.00	1.00	
Occupation	Student	41.9 (49)	5.58 (2.95–10.56)	1.95 (0.53–7.18)	0.316
	Farming	21.8 (26)	2.17 (1.10–4.27)	1.73 (0.75–3.96)	0.195
	Business	18.8 (25)	1.79 (0.91–3.54)	1.60 (0.68–3.77)	0.281
	Salary earner	11.4 (16)	1.00	1.00	
Awareness	Aware	27.8 (45)	1.49 (0.97–2.30)	1.73 (1.02–2.95)	0.043
	Not aware	20.5 (71)	1.00	1.00	
Distance from stream (metres)	≤ 100	27.7 (91)	2.37 (1.46–3.86)	1.19 (0.63–2.26)	0.584
	˃ 100	13.9 (25)	1.00		
Degree of water contact	High^a^	37.8 (54)	1.67 (1.05–2.65)	1.23 (0.71–2.12)	0.455
	Low^b^	26.7 (52)	1.00	1.00	

CI = confidence interval, OR = odds ratio, ^#^OR = adjusted OR using multivariate regression analysis

^a^ activities include; bathing, laundry and washing of motorbike and increased frequency to stream (≥ Thrice/week).

^b^ activities include; fetch water and farming and reduced frequency to stream (< thrice/week).

**Fig 3:**
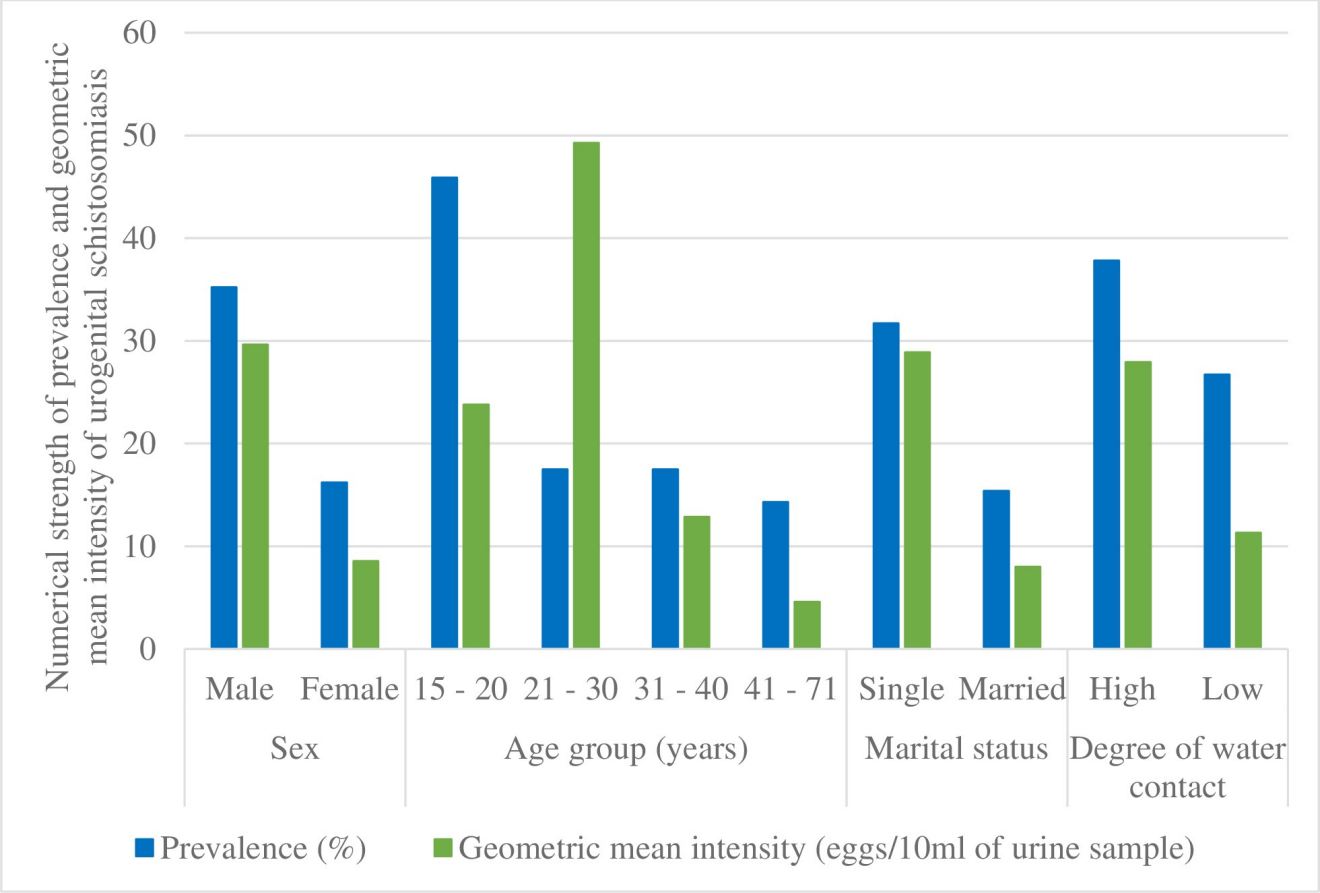
Bar chart showing the prevalence and geometric mean intensity of *S*. *haematobium* infection with respect to sociodemographic characteristics and stream contact behaviour.

Similarly, higher mean intensity of egg excretion were recorded in males (29.62 eggs per 10ml), singles (28.89 eggs per 10ml), and the age groups 15–20 (23.79 eggs per 10ml) and 21–30 years (49.28 eggs per 10ml). The difference was significant Table 4. There was a significant negative correlation (r = -0.228; p = 0.014) between age and egg output. Though not significant, higher mean intensities of the infection were recorded in respondents who had high degree of contact with stream (27.93 eggs per 10ml) Fig 3.

**Table 4:** Intensity of *S*. *haematobium* infection based on sociodemographic characteristic.

Variable	Category	Intensity of infection		Geometric mean egg count
Gender		**Light % (n)**	**Heavy % (n)**	
	Male	38.3 (36)	25.5 (24)	29.62
	Female	31.9 (31)	4.3 (4)	8.55
	p-value	0.003		0.009^**#**^
Age group(years)	15–20	31.9 (31)	14.9 (14)	23.79
	21–30	7.4 (7)	9.6 (9)	49.28
	31–40	17.0 (16)	5.3 (5)	12.86
	41–71	13.8 (13)	0 (0)	4.58
	p-value	0.010		0.019^**$**^
Educational level	At least secondary	47.9 (45)	21.3 (20)	20.62
	At least primary	22.3 (21)	8.5 (8)	15.08
	p-value	0.755		0.494^**#**^
Marital status	Single	41.5 (39)	24.5 (23)	28.89
	Married	28.7 (27)	5.3 (5)	7.99
	p-value	0.031		0.004^**#**^
Distance from stream (metres)	≤ 100	56.4 (53)	24.5 (23)	19.77
	˃ 100	13.8 (13)	5.3 (5)	14.89
	p-value	0.836		0.768^**#**^
Degree of water contact	High^a^	62.3 (33)	37.7 (20)	27.93
	Low^b^	81.0 (34)	19.0 (8)	11.32
	p- value	0.008		0.073^**#**^

^$^P-value obtained using Kruskal Wallis test

^#^ P-value obtained using Mann-Whitney test.

^a^ activities include; bathing, laundry and washing of motorbike and increased frequency to stream (≥ Thrice/week).

^b^ activities include; fetch water and farming and reduced frequency to stream (< thrice/week).

#### Stream contact behaviour in association with gender and age

The results displayed in Table 5 show that the level of exposure to infested water differed significantly among gender and age groups. Males indulged in more water contact activities for bathing, fetching of water, farming and washing motorbikes which entailed longer duration in the stream whereas females were involved more with the stream for domestic purposes. Accordingly, the frequency of water contact between males and females was significantly different (P < 0.001). A higher number of visits (≥ thrice/week) to the stream were recorded among males than females. Compared with the older age groups, the younger age group (15–20 years) was significantly (p <0.001) associated with more stream activities. Nonetheless, the difference in the degree of contact with stream among age groups was not significant Table 5.

**Table 5:** Stream contact behaviour in association with gender and age.

Stream contact behaviour	Gender (%)		Age group (years) (%)			
Distance to stream (metres)	Male	Female	15–20	21–30	31–40	41–71
≤ 100	67.6	63.1	73.4	64.9	58.8	64.3
˃ 100	32.4	36.9	26.6	35.1	41.3	35.7
χ^2^; P value	1.043; 0.307		6.094; 0.107			
Stream usage						
Yes	80.1	59.2	81.7	61.4	57.5	69.0
No	19.9	40.8	18.3	38.6	42.5	31.0
χ^2^; P value	22.663; <0.001		18.718; <0.001			
Stream activity						
All three	18.4	2.0	13.5	7.1	7.6	6.9
At most two	34.0	16.2	32.6	24.3	10.9	27.6
Bathing	17.7	16.8	13.5	15.7	21.7	17.2
Domestic chore	22.0	52.3	39.3	44.3	44.6	31.0
Farming	7.8	12.7	1.1	8.6	15.2	17.2
χ^2^; P value	56.850; < 0.001		30.548; 0.002			
Frequency to stream per week						
≥ Thrice	69.5	51.3	65.2	57.1	50.0	63.2
< Thrice	30.5	48.7	34.8	42.9	50.0	36.8
χ^2^; P value	11.286; 0.001		5.213; 0.157			
Degree of water contact						
High^a^	53.2	34.5	49.4	38.6	34.8	46.0
Low^b^	46.8	65.5	50.6	61.4	65.2	54.0
χ^2^; P value	11.741; 0.001		4.869; 0.182			

χ^2^ = Pearson Chi-square test.

^a^ activities include; bathing, laundry and washing of motorbike and increased frequency to stream (≥ Thrice/week).

^b^ activities include; fetch water and farming and reduced frequency to stream (< thrice/week).

## Discussion

The endemicity of UGS presents an enormous public health challenge in the South West Region, Cameroon, where a new unmapped transmission focus has been established in peri-urban settings such as the Tiko health area [20]. In this context, this study reports on the current epidemiological status of UGS among reproductive age individuals in the THA. Our results confirmed *S*. *haematobium* transmission in the semi-urban area of Tiko, Mount Cameroon area with the occurrence of infection at 22.8% among reproductive age individuals, with higher risk and severity of infection seen more in younger adults and males than in their respective counterparts.

It is obvious that the study area is meso-endemic (prevalence 20–40%) for urogenital schistosomiasis [35]. This prevalence level is within the range of infection rates (14.9–33.0%) reported between 2016–2019 among the reproductive age population in some villages; Munyenge (Wenpje *et al*. [21] (22.3%); Ndassi *et al*. [22] (14.9%), and Ikata, Likoko area (Ebai et al., [18] (33%)) in the Bafia Health area. Human UGS appears to be highly endemic in peri-urban/rural areas and closely associated with low socioeconomic status [36]. Transmission of *S*. *haematobium* varies between countries, between regions in the same country and even between seasons. Considerably, lower prevalence of UGS have been reported from studies conducted in urban settings in other parts of Africa such as Bamako, Mali (14.7%) [37] and Northern Ivory Coast (1.9%) [38]. Several environmental and ecology factors may explain differences in the transmission intensity of schistosomiasis [39]. The Mount Cameroon area has an equatorial forest climate whereas Bamako and Northern Ivory Coast are characterised by dry Sahelian conditions [37, 38]. Ndassi *et al*., [22] reported a lower prevalence of UGS of 14.9% among adults in a study carried out in the Bafia health area during the dry season.

The THA is close to some villages, such as Munyenge, found in the Bafia health area, Mount Cameroon area. Bio-ecologically, both health areas are characterized by favourable environmental conditions for the *Bulinus* snail intermediate host reproduction and parasite survival in the springs and streams [21, 40]. The hydrology of communities in the Mount Cameroon area consist of networks of streams [41] and possibly snail host are being washed down stream to neighbouring towns. For the establishment of schistosomiasis in new transmission foci, the bionetworks and environmental conditions, appropriate aquatic snail intermediate hosts, and the human definitive host must converge in space and time in suitable water bodies [37]. In the case of Tiko, population movement into the suburban areas of endemic foci, the increasingly dependence on stream water for livelihood and lack of basic sanitation may have resulted in urine contamination of the streams with consequent infection of intermediate host and emergence of the new focus for UGS transmission in the Mount Cameroon area.

The factors, which were predictive of infection in the present study were age and gender. Individuals in the age group 15–20 years and 31–40 years were five and two times respectively more likely at risk of *S*. *haematobium* infection. In most schistosome infection surveys, age is seen as a major determinant of *S*. *haematobium* infection [42–44]. The younger age individuals (15–20years) are more involved with the open water sources for bathing, laundry and collection of water for domestic purposes. These findings corroborate previous reports in the Mount Cameroon area [19], elsewhere in Cameroon [42, 45], as well as other parts of Africa [43, 46]. Age-acquired immunity to reinfection contributes to declining trend in infection prevalence with increasing age [46]. This may explain the low infection status in the older age group of 41–71 years despite their intense water contact behaviours which include bathing, domestic chores and farming. Surprisingly, the odds of infection were higher in the age group 31–40 when compared with 21-30-year olds. Besides bathing and domestic chores, the streams also served as a source of water for washing of motorbikes and farming among the 31–40 years age group. In some neighbourhood with shortage in water supply, accessing and using stream water is vital not only for household livelihood but also for agricultural purpose such as washing of pumpkin seeds. The lack of bridges in certain parts of the area, compels the population to cross these streams to their farms and other areas within the municipality exposing them to cercariae infested water. The intensity of egg excretion was significantly higher (p = 0.012) in the younger aged individuals age when compared with the older adults. The inverse relationship between egg load and age supports the well documented observation of immune-mediated reduction of worm fecundity with host age in *S*. *haematobium* infection [47, 48].

As expected, the male gender had significantly higher prevalence (35.2% Vs 16.2%) and geometric mean intensity of infection (29.62 Vs 8.55) than females. Several studies have reported that schistosomiasis is a disease that affects males more frequently [49–54], which our study also showed that these individuals have a significantly higher chance (OR = 2.6) of acquiring the infection. This finding is probably related to the greater involvement of men in activities in streams near the community. In contrast, a significantly higher prevalence of schistosomiasis has been reported among females than males in some studies, which is attributed to frequent contact with water in the execution of domestic chores including fetching of water and laundry [45, 55]. In this study, males had higher degree of contact with stream, specified by activities which entailed longer duration in the stream (bathing, and washing of motorbike) thus increasing their risk of exposure to infection. Previous findings have shown that increase in intensity of infection with urogenital schistosomiasis increased the risk of FGS. Equally, the high infection intensity observed in males in the study area may be associated with the risk of MGS. Infection can cause genital ulcers and other lesions [13], induce pathology of the seminal vesicles and the prostate with sexual dysfunction and irreversible long-term reproductive health consequences including infertility [14]. Also, with high infection intensities, eggs of schistosomes can became trapped in tissues of the liver, spleen, kidney and peritoneum with severe and complex pathological consequences which may degenerate to late-stage sequelae [54, 56].

Our findings showed that individuals aware of UGS were 1.73 times more likely to be infected compared with individuals who were not aware. Similar results have been reported in Cameroon [57], Nigeria [58] and in South Africa [59]. Notably, the majority of individuals resident in communities’ located ≤ 100m around infested water sources were aware of UGS possibly through exposure to the disease indicators. Haematuria and *Bulinus* spp. is used as a strong indicator of awareness about urogenital schistosomiasis. The strong association between awareness and disease clearly suggests that infected respondents most likely came across the snail intermediate host while bathing, fishing, and/or fetching water in infested streams [58]. Moreover, this finding may indicate the poor knowledge on UGS transmission and prevention. Even so, the knowledge gained might not be enough to protect these communities from infection as the lack of access to safe drinking water and adequate sanitation are the driving forces behind the risk behaviour of individual community members [60]. This poses serious implication in the control and treatment of the infection in this area. In Tiko, inadequate or shortage of portable water supply may contribute to high dependence on open water sources (66.4%) given that 87.6% of the respondents indicated access to some form of pipe-borne water. In line with reports of other studies [19, 61], no socioeconomic variable was independently associated with UGS prevalence in the study area suggesting that improving socioeconomic status alone may not contribute to a significant reduction of schistosomiasis prevalence rate in these communities. In this regard, health talks, to raise community awareness for better understanding of the social and behavioural determinants that influence disease transmission is recommended as a first line of action. The extension and functionality of piped water sources and the construction of car wash points in this endemic area will reduce the need for intense contact with infested water particularly among males and younger age individuals thus decreasing the transmission of UGS in this area.

### Limitations of the survey

The analyses of these data of this study depended on only one urine specimen per participant. WHO encourages the collection of three urine samples per participants for accurate detection of infection. The collection of multiple samples also enables the assessment of intra and inter- specimen variation of the egg output [61]. Although researchers were cognizant of these facts, it was difficult to obtain multiple samples from participants. The participants complained of lack of time and some were embarrassed of handling bodily specimens in public. In spite of this, our findings provide valuable evidence required for evidence-based decision making.

## Conclusion

The prevalence obtained in this study shows that Tiko is a moderate-risk area for *S*. *haematobium* transmission. Younger age group (15–20years), and male gender comprise the determinant factors associated with increased risk of *S*. *haematobium* infection. Males may be predisposed to higher risk of disease severity than females. Awareness of the disease does not prevent individuals in this study area from using cercariae infested streams but the provision of public infrastructures to limit contact with streams will curb the transmission and morbidity associated with urogenital schistosomiasis. However, a productive and sustainable intervention cannot be achieved without adequate education.

## Supporting information

S1 Checklist Questionnaire. (PDF)
