# Supplementary material for: The epidemiological status of urogenital schistosomiasis among reproductive aged individuals in the Tiko Health Area- a semi-urban setting in the Mount Cameroon area
Source: PLoS Negl Trop Dis. 2021 Jan 11;15(1):e0008978. doi: 10.1371/journal.pntd.0008978 (PMC7822554; doi:10.1371/journal.pntd.0008978)
Supplement: S1 Checklist — (PDF) [file pntd.0008978.s001.pdf]

QUESTIONNAIRE

A. PARTICIPANT'S IDENTITY

Code ____________________ Telephone Number ____________________

1. Sex: male: ______ Female: ________ 2. Age: ________ (years)
3. Area of residence: ____________________

B. DEMOGRAPPHIC INFORMATION

1. Level of educational attainment. Primary ☐ Secondary ☐ Tertiary ☐
2. Marital status. Married ☐ Single ☐ Divorced ☐ Widow ☐
3. Occupation. Farming ☐ Business ☐ Student ☐ Others (specify) ____________

C. HISTORY OF UROGENITAL SCHISTOSOMIASIS

4. How long have you live here? ____________________
5. Are you menstruating (for female)? Yes ☐ No ☐
6. Do you experience any pain when urinating? Yes ☐ No ☐
7. When you urinate is there blood in your urine? Yes ☐ No ☐
8. Have you ever heard of urogenital schistosomiasis? Yes ☐ No ☐
9. Have you been diagnosed of urogenital schistosomiasis before? Yes ☐ No ☐
10. When was the last time you took praziquantel? ____________________

D. STREAM CONTACT BEHAVIOUR

11. a) Do you have tap/potable water in this community? Yes ☐ No ☐

    b) If yes in (a) above what is the source from which it is taped? a) Treated source b) stream

    c) If yes in (a) above, is the tap always flowing? Yes ☐ No ☐
12. Where do you fetch water for domestic use? a) Stream b) Tap c) Both

13. What is the distance from your house to the stream? ----------------- (m)

14. What are the activities you carry out at the Stream? Bathing ▭ Laundry ▭

Fetch water ▭ Washing pumpkin seeds (egusi) ▭ Washing motorbike ▭

Crossing ▭

15. How often do you go to the stream per week? Once ▭ Twice ▭ Thrice ▭

More than thrice ▭
